# Signaling mechanisms and behavioral function of the mouse basal vomeronasal neuroepithelium

**DOI:** 10.3389/fnana.2014.00135

**Published:** 2014-11-26

**Authors:** Anabel Pérez-Gómez, Benjamin Stein, Trese Leinders-Zufall, Pablo Chamero

**Affiliations:** Department of Physiology, University of Saarland School of MedicineHomburg, Saarland, Germany

**Keywords:** vomeronasal organ, olfaction, Gαo signaling, V2R, peptides, pheromone, behavior

## Abstract

The vomeronasal organ (VNO) is a sensory organ that is found in most terrestrial vertebrates and that is principally implicated in the detection of pheromones. The VNO contains specialized sensory neurons organized in a pseudostratified neuroepithelium that recognize chemical signals involved in initiating innate behavioral responses. In rodents, the VNO neuroepithelium is segregated into two distinct zones, apical and basal. The molecular mechanisms involved in ligand detection by apical and basal VNO sensory neurons differ extensively. These two VNO subsystems express different subfamilies of vomeronasal receptors and signaling molecules, detect distinct chemosignals, and project to separate regions of the accessory olfactory bulb (AOB). The roles that these olfactory subdivisions play in the control of specific olfactory-mediated behaviors are largely unclear. However, analysis of mutant mouse lines for signal transduction components together with identification of defined chemosensory ligands has revealed a fundamental role of the basal part of the mouse VNO in mediating a wide range of instinctive behaviors, such as aggression, predator avoidance, and sexual attraction. Here we will compare the divergent functions and synergies between the olfactory subsystems and consider new insights in how higher neural circuits are defined for the initiation of instinctive behaviors.

## Introduction

The mammalian olfactory system is composed of multiple chemosensory subsystems that differ in anatomical location, receptor types, and innervation within the central nervous system (Munger et al., 2009). The vomeronasal organ (VNO) is the sensory substructure of the accessory olfactory system that is specialized in the identification of specific chemosensory cues important for the display of socio-sexual behaviors. The VNO detects a range of molecules that can be both volatile and non-volatile, including peptides and small proteins. These molecules may be either pheromones secreted externally by conspecifics in urine, tears and saliva, as well as non-pheromones such as those from preys and predators (Wyatt, 2014). During olfactory investigation, chemosignals entering the nasal cavity are pumped into the VNO lumen, where they are detected by vomeronasal sensory neurons (VSNs). The mechanism of pumping consists in a distension-contraction of vascularized erectile tissue located in the lateral side of the VNO lumen (Trotier, 2011). Sympathetic stimulation triggers this vascular pump (Ben-Shaul et al., 2010) during exploratory behaviors, probably as a result of detection of other volatile stimuli by the main olfactory system (Martínez-García et al., 2009). Therefore, vomeronasal activity is unlikely to remain as an autonomous olfactory unit but instead requires intense interaction with other sensory inputs to transduce the stimulus information to downstream targets. Mature VSNs reside in the medial side within a pseudostratified sensory neuroepithelium formed by bipolar neurons, directed to the aqueous lumen, with an extended dendrite and cilia in which detection takes place. The VNO emerges in evolution on amphibians during adaptation to life on land (Trotier, 2011), and is present in many but not all mammals. It is missing in cetaceans, some bats and some primates (Mucignat-Caretta, 2010). In humans it appears to be vestigial (Trotier et al., 2000; Meredith, 2001). In the mouse, the VNO neuroepithelium is divided in non-overlapping apical and basal layers that express two different families of receptors, vomeronasal receptors 1 and 2 (V1Rs and V2Rs), respectively (Dulac and Axel, 1995; Herrada and Dulac, 1997; Matsunami and Buck, 1997; Ryba and Tirindelli, 1997). Furthermore, a subset of neurons in the VNO express five members of the formyl peptide receptor (FPR) family, four of them apical and one basal (Liberles et al., 2009; Riviere et al., 2009). The wiring logic of the VSN projection is maintained at the level of the accessory olfactory bulb (AOB): Apical VSNs connect with glomeruli in the rostral half of the AOB, whereas basal VSNs axons synapse in the caudal AOB. The implications of this neuronal segregation in the accessory olfactory system for the processing of chemosensory information are largely unknown. Moreover, certain species such as the goat, marmoset and tammar wallaby lack this anatomical segregation and display uniform-type vomeronasal epitheliums (Takigami et al., 2000, 2004; Schneider et al., 2012). Recent advances in the identification of subzone-specific ligands and ablation of sensory transduction components have now enabled a more detailed analysis of the molecular mechanisms controlled by each neural VNO pathway to initiate olfactory-mediated behaviors.

## Molecular and functional organization of the basal VNO

The spatial segregation in the VNO correlates with the differential expression of two G-protein subunits, Gαi2 and Gαo (Berghard and Buck, 1996; Jia and Halpern, 1996). These G-proteins are the initial step of a phospholipase C (PLC)-mediated signaling cascade to transduce sensory signals detected by V1Rs and V2Rs (Chamero et al., 2012). In the VNO, G-proteins form complexes identified as Gαoβ2γ8 in the basal and Gαi2β2γ2 in the apical neurons (Montani et al., 2013). The functional importance of the G-protein subunits in mediating sensory transduction responses was established by ablating genes in mice. VSNs from Gαo mutants display severe deficits to transduce chemosensory signals that result in a number of behavioral alterations including reduced olfactory-mediated aggression (Chamero et al., 2011). Furthermore, Gαo seems to be critical for the maintenance of the cellular homeostasis in the postnatal sensory neuroepithelium as Gαo mutant mice show a remarkable reduction in the size of the basal neuronal layer (Tanaka et al., 1999). Likewise, mutant mice lacking Gγ8 subunit display a similar cell loss in the VNO epithelium and a diminished aggressive response (Montani et al., 2013). Thus, Gαo and subsequent coupling with Gβ2γ8 represent the key candidate molecules to control PLC activation through specific olfactory stimuli in the basal VNO (Rünnenburger et al., 2002).

PLC activation produces inositol 1,4,5-trisphosphate and diacylglycerol, the only known activator of a member of the transient receptor potential family of ion channels, Trpc2. Trpc2 expressed in both apical and basal VNO layer is another key player in VNO signal transduction (Liman et al., 1999). Genetic ablation of *Trpc2* results in dramatic consequences in vomeronasal function in terms of VSNs responsiveness to urinary signals, cell survival, and socio-sexual behavior (Leypold et al., 2002; Stowers et al., 2002; Kimchi et al., 2007; Ferrero et al., 2013; Wu et al., 2014). The *Trpc2* gene, initially assumed to be exclusively expressed in the VNO, has been abundantly detected in the main olfactory epithelium (MOE) as well (Omura and Mombaerts, 2014). Therefore, the contribution of MOE-specific Trpc2 signaling to the described behavioral *Trpc2* null phenotype remains to be dissected. This may help to explain observed phenotypic discrepancies of *Trpc2* deletion (Leypold et al., 2002; Stowers et al., 2002; Kimchi et al., 2007) and surgical VNO removal (Clancy et al., 1984; Wysocki and Lepri, 1991; Pankevich et al., 2004; Martel and Baum, 2009) on ultrasonic vocalizations and sex-specific behaviors in both male and female mice. Additional signaling components expressed in both basal and apical VSNs are the discovered calcium-activated chloride and potassium channels, which seem to participate in the VNO sensory responses (Dibattista et al., 2008; Billig et al., 2011; Kim et al., 2011, 2012). A recent study using deep RNA sequencing identified nearly 800 novel, putative protein-coding, multi-exonic genes expressed in the whole VNO (Ibarra-Soria et al., 2014). Thus, new vomeronasal signaling components are expected to emerge in the near future.

**Figure 1:**
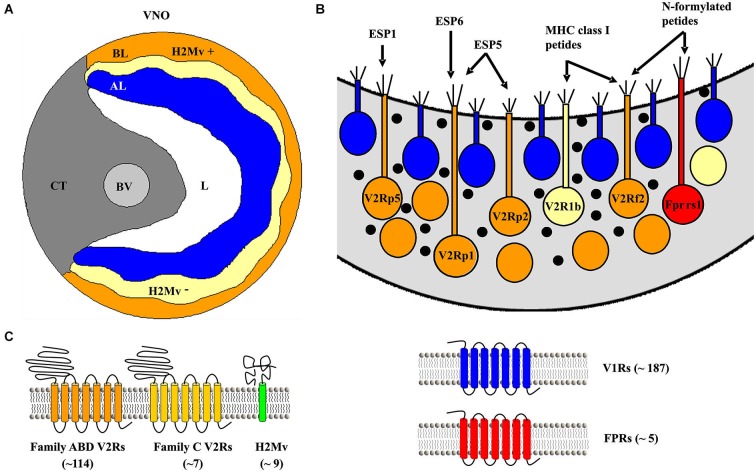
**The mouse vomeronasal organ (VNO) and established receptors located in the epithelium. (A)** Schematic coronal section of the VNO. AL, apical layer of the sensory epithelium (*blue*); BL, basal layer (*yellow/orange*); BV, blood vessel; CT, cavernous tissue; *H2Mv*+, sensory epithelium cells lacking one of the nine known *H2-Mv* genes; *H2Mv*−, sensory neurons not expressing any of the nine *H2-Mv* genes; L, lumen. **(B)** Schematic drawing of sensory neurons showing their location in the basal sensory epithelium with their proposed ligands. **(C)** Schematic picture of the proposed receptors expressed in the basal part of the VNO sensory epithelium.

The range and specificity of chemosignals detected by the VNO depend on the expression of particular vomeronasal receptors. Three families of vomeronasal receptor genes have been identified in the mouse VNO: V1Rs, V2Rs (also known as *Vmn1rs* and *Vmn2rs*) and *Fprs* (Tirindelli et al., 2009). VSNs in the basal layer of the VNO express V2Rs as well as a single FPR member, Fpr-rs1. The mouse genome contains 121 functional V2R genes and—curiously—even a larger number (158) of pseudogenes (Young and Trask, 2007). V2Rs evolved independently from V1Rs and differ in the type of chemosignals they detect, to date: peptides/proteins by V2Rs and small organic molecules by V1Rs, and in the expression logic: VSNs expressing V1Rs show a single-receptor type expression whereas basal VSNs expresses one V2R member of the subfamily C, along with an additional V2R gene from subfamily A, B or D in a non-random manner (Figure 1; Martini et al., 2001; Silvotti et al., 2007; Ishii and Mombaerts, 2011). Until now, only a handful of V2Rs have been deorphanized (Table 1) and all of them belong to the subfamily A, which represents nearly 85% of the V2R genes. Furthermore, V2R sequences of inbred mouse strains show high variation in subfamily A1, A5 and A8 while subfamilies B, C and D are highly conserved (Wynn et al., 2012). The importance of V2R subfamily expression for VSN pheromone specificity and detection still needs to be resolved. However, recent evidences suggest that expression of multiple receptors may have a role in the combinatorial activation logic of VSNs by overlapping specificities and concentrations (Leinders-Zufall et al., 2009; Kaur et al., 2014).

**Table 1:** **List of signaling molecules with proposed receptors located in the basal sensory epithelium**.

Chemosignal	Source	Receptor	Gαo need	Behavioral effects	References
ESP1	Male mouse tears	V2Rp5 (Vmn2r116)	✔	-Lordosis	Kimoto et al. (2007), Haga et al. (2010)
ESP5	Mouse tears	V2Rp1 (Vmn2r112), V2Rp2 (Vmn2r111)	?	?	Kimoto et al. (2007), Dey and Matsunami (2011)
ESP6	Mouse tears	V2Rp1 (Vmn2r112)	?	?	Kimoto et al. (2007), Dey and Matsunami (2011)
ESP22	Juvenile mouse tears	?	?	-Inhibition of male sexual behavior	Ferrero et al. (2013)
HMW/MUPs	Mouse urine	?	✔	-Male-male aggression	Chamero et al. (2007, 2011)
				-Maternal aggression	Martín-Sánchez et al. (2014)
		?	✔	-Preference in females	Hurst et al. (2001), Cheetham et al. (2007), Sherborne et al. (2007), Roberts et al. (2010)
				-Urine countermarking behavior	Kaur et al. (2014)
				-Puberty acceleration	Mucignat-Caretta et al. (1995)
				-Ovulation	Morè (2006)
MUP3		?	✔	-Male-male aggression -Countermarking	Kaur et al. (2014)
MUP20		?	✔	-Attraction in females -Conditioned place preference -Countermarking behavior -Maternal aggression	Roberts et al. (2010, 2012), Kaur et al. (2014), Martín-Sánchez et al. (2014)
LMW	Mouse urine	?	*	-Male-male aggression -Maternal aggression	Chamero et al. (2007, 2011)
MHC class I peptides	Mouse urine	V2R1b (Vmn2r26) V2Rf2 (Vmn2r81)	✔	-Bruce effect	Leinders-Zufall et al. (2009, 2014)
N-formylated peptides	Bacteria or mitochondria	Fpr-rs1, V2Rf2 (Vmn2r81)	✔	?	Liberles et al. (2009), Riviere et al. (2009), Bufe et al. (2012), Leinders-Zufall et al. (2014)

✔: Gαo expression needed to detect the chemosignal; *: partial detection by both apical and basal layers; ?: not determined; Brackets: alternative receptor names.

In addition to V2R expression, a subset of basal VSNs have been shown to express genes of the major histocompatibility complex (MHC) class 1b, also known as *H2Mv* molecules (Ishii et al., 2003; Loconto et al., 2003). This family comprises nine genes—M1, M9, M11 and six members of the M10 family—clustered in the genome. Most of the neurons express a single gene, but some seem to be able to express two or three. The proteins localize to the dendritic tips and microvilli of VSNs predicting a potential role in pheromone detection or signal transduction. *H2Mv* molecules have been proposed to form a protein complex together with V2Rs and β2-microglobulin necessary for the transport of the receptor to the plasma membrane (Loconto et al., 2003). Certainly, *H2Mv* molecules are dispensable for chemosignal detection but seem to be required to show high sensitivity to peptide ligands necessary for the display of aggressive and sexual behaviors (Leinders-Zufall et al., 2014). A fraction of Gαo-expressing VSNs do not co-express *H2-Mv* genes, for example sensory neurons expressing the Vmn2r26 receptor (also known as V2r1b), which are localized to the upper sublayer of the basal VNO. This spatial segregation is also maintained at the level of the AOB, defining a tripartite organization of the mouse vomeronasal system (Figure 1; Ishii and Mombaerts, 2008).

A third population of Gαo-expressing VSNs expresses Fpr-rs1 (Figure 1), an additional chemosensory G-protein coupled receptor (GPCR) that belongs to the FPR family (Liberles et al., 2009; Riviere et al., 2009). Fpr-rs1 neurons do not co-express V2Rs or other FPR members. Fpr-rs1 was found to display stereo-selectivity for peptides with a D-amino acid in the C-terminal position, which are contained in pathogenic microorganisms (Bufe et al., 2012). This ligand detection profile raises the possibility of a pathogenic sensing role of the vomeronasal system to assess the health status of conspecifics during social communication.

## Sensory ligands detected by the basal VNO

Early functional experiments in the rat vomeronasal sensory epithelium described urine profile activation differences between the apical and basal VNSs (Inamura et al., 1999). Up to now, considerable evidence has shown that sensory neurons located in the basal VNO detect several families of nonvolatile peptide and protein chemosignals. The family of MHC class I peptides were the first identified sensory stimuli for V2R-positive VSNs (Leinders-Zufall et al., 2004). MHC peptides, that have been identified in mouse urine together with some other interesting species-specific peptide ligands (Sturm et al., 2013), are detected by VSNs at ultralow concentrations and require Gαo, but not Trpc2 (Leinders-Zufall et al., 2004; Kelliher et al., 2006; Chamero et al., 2011). Detection of MHC peptide ligands does not require or correlate with the expression of *H2Mv* molecules. Instead, vomeronasal receptors seem to be essential: genetic deletion experiments showed two V2Rs—Vmn2r26 (V2R1b) and Vmn2r81 (V2Rf2)—which are needed by their VSNs to respond to specific MHC peptides (Leinders-Zufall et al., 2009, 2014). Interestingly, MHC-independent peptides as well as formylated and non-formylated versions of mitochondrial peptides can also activate V2R positive VSNs (Sturm et al., 2013; Leinders-Zufall et al., 2014).

A second family of peptides—the exocrine-gland-secreting peptide (ESP) family—has been identified to be detected by V2R-expressing VSNs. The mouse genome contains 24 members of this family of 5–15 kDa peptides expressed in extraorbital, lacrimal, Harderian, and submaxillary glands (exocrine glands) in a sex- and strain-specific manner (Kimoto et al., 2007). Field potential recordings have shown that at least 16 ESPs elicit electrical responses in the VNO (Kimoto et al., 2007; Haga et al., 2010; Ferrero et al., 2013). Responses to ESP1, 5 and 6 have been linked with expression of a specific V2R subfamily (V2Rp) either by c-Fos activity measures, or by heterologous expression (Haga et al., 2010; Dey and Matsunami, 2011).

A third group of nonvolatile chemosignals functioning as stimuli of the basal VNO layer consist of the major urinary proteins (MUPs) and other related lipocalins. MUPs are abundantly expressed in urine, but are also found in other secretions, including saliva, milk, and even the olfactory epithelium (Ibarra-Soria et al., 2014). In the mouse, MUPs are encoded by a multigene family of 21 homologous, highly identical genes which are expressed in a sex- and strain-dependent manner (Logan et al., 2008; Mudge et al., 2008). MUPs evoke Ca^2+^ and electrophysiological responses on Gαo- and V2R-expressing VSNs using Trpc2/Gαo signaling (Chamero et al., 2007, 2011) and benefit of the presence of *H2Mv* molecules (Leinders-Zufall et al., 2014), but specific MUP receptors are yet to be described. Mouse VSNs detect conspecific MUPs utilizing a combinatorial strategy (Kaur et al., 2014) in addition to being activated by orthologous MUP proteins secreted by cats and rats (Papes et al., 2010), adopting a new chemosensory role as interspecific genetically encoded signals.

## Behavioral responses

Odor-driven behaviors are reported to depend on the basal VNO layer largely relying on two main criteria: First, as result of gene knockout studies of specific signal transduction molecules or receptors from basal VSNs, and/or second, from experiments using chemosignals shown to (specifically) activate basal VSNs. Aggressive behavior toward intruder males was identified to require sensory transduction from basal VSNs. Ablation of either Gαo, Gγ8, and *H2Mv* genes severely reduced or eliminated male-male and maternal aggression (Chamero et al., 2011; Montani et al., 2013; Leinders-Zufall et al., 2014), both types of aggression shown to be partially elicited by MUPs (Chamero et al., 2007, 2011; Kaur et al., 2014). Gαo gene removal also resulted in a wide range of deficient reproductive behaviors in female mice, including defective puberty acceleration (Vandenbergh effect) and estrus induction (Whitten effect) in adult mice (Oboti et al., 2014). The identities of the pheromones that underlie the Vandenbergh and Whitten effects are still controversial. Molecules that activate either apical (Jemiolo et al., 1986; Novotny et al., 1999) and basal (Nishimura et al., 1989; Mucignat-Caretta et al., 1995; Morè, 2006) VSNs have been described to participate in these estrus-modulating effects. Nonetheless, it cannot be excluded that multiple olfactory subsystems are required to evoke certain behavioral responses triggered by odorant blends. Consistent with this view, apical and basal VNO subsystems are necessary and seem to interact in the generation of male and female aggression (Del Punta et al., 2002; Norlin et al., 2003; Chamero et al., 2011). In contrast, other pheromone-induced behavioral responses are controlled by single VNO receptor-ligand interactions. The sexual stance lordosis is enhanced by the tear peptide ESP1 that activates Vmn2r116 receptor, and mutant animals lacking this receptor display a striking lordosis deficit (Haga et al., 2010). Consistent with these experiments, surgical lesions on the VNO and AOB (Keller et al., 2006; Martel and Baum, 2009) as well as deletion of Gαo and *H2Mv* genes (Leinders-Zufall et al., 2014; Oboti et al., 2014) also resulted in a drastic reduction of lordosis. Another member of the ESP peptide family has been implicated in the control of a different type of sexual behavior: ESP22, expressed in tears of prepubertal mice, was found to elicit a Trpc2-dependent inhibitory effect on adult male mating behavior (Ferrero et al., 2013).

MHC peptides have been shown to alter female reproductive function as detected in the Bruce effect test (Leinders-Zufall et al., 2004). Here, pregnancy is terminated in a recently mated female by the odor of a strange male. This test is frequently used as paradigm to assess genetic compatibility and individual recognition. MUPs are also proposed to operate as olfactory cues governing individual recognition, as they are genetically encoded and highly polymorphic (Cheetham et al., 2007). Hence, MUPs have been reported to mediate inbreeding avoidance, countermarking and female sexual attraction (Hurst et al., 2001; Sherborne et al., 2007; Roberts et al., 2010; Kaur et al., 2014). Related to this recognition capacity, MUP detection also plays an important role on aggression, conditioned learned spatial preference and detection of predators (Chamero et al., 2007, 2011; Papes et al., 2010; Roberts et al., 2012). Remarkably, single MUP ligands are able to evoke multiple behavioral responses depending on the gender and reproductive status of the receiving individual; MUP20—also known as darcin—may elicit sexual attraction and spatial learning in estrous females, maternal aggression in lactating females, and countermarking and aggression in adult males (Roberts et al., 2010, 2012; Kaur et al., 2014; Martín-Sánchez et al., 2014). Whether these responses are mediated by a single or multiple sensory neurons or receptor types remain to be elucidated.

These recent advances in the identification of specialized receptors, neural pathways and sensory ligands from the basal VNO layer provide the tools to stimulate, study, and determine the molecular mechanisms that trigger specific behavioral responses.

## Conflict of interest statement

The authors declare that the research was conducted in the absence of any commercial or financial relationships that could be construed as a potential conflict of interest.
